# Environmental DNA (eDNA) contamination patterns of African swine fever virus (ASFV) in swine transport vehicles in the Philippines

**DOI:** 10.17221/84/2024-VETMED

**Published:** 2025-05-28

**Authors:** Mariane Jane Bartolome, Laurie Ann Marcella Aguirre, Caressa Marielle Poliquit, Iahleah Besas, Jorge Gil Angeles, Jomar Rabajante, Sherwin Camba, Fletcher Del Valle, Adelberto Ambrocio, Flomella Caguicla, Mary Grace Bustamante, Dennis Umali

**Affiliations:** ^1^Livestock Research and Development Division, Bureau of Animal Industry, Department of Agriculture, Quezon City, Philippines; ^2^Department of Veterinary Clinical Sciences, College of Veterinary Medicine, University of the Philippines Los Baños (UPLB), Laguna, Philippines; ^3^Institute of Biological Sciences, College of Arts and Sciences, UPLB, Laguna, Philippines; ^4^Philippine Genome Center – Program for Agriculture, Livestock, Forestry and Fisheries, UPLB, Laguna, Philippines; ^5^Institute of Mathematical Sciences, College of Arts and Sciences, UPLB, Laguna, Philippines; ^6^Institute of Animal Science, College of Agriculture and Food Sciences, UPLB, Laguna, Philippines; ^7^Office of the Provincial Veterinarian, Provincial Government of Quezon, Pagbilao, Philippines; ^8^Office of the Provincial Veterinarian, Provincial Government of Laguna, Sta Cruz, Laguna, Philippines

**Keywords:** African swine fever, biosecurity, environmental surveillance, fomites, qPCR

## Abstract

Since its introduction in 2019, African swine fever (ASF) has spread to all regions of the Philippines, affecting 73 out of its 82 provinces. To assess the environmental DNA (eDNA) contamination patterns of the ASF virus (ASFV) in swine transport vehicles and evaluate its measures of association, a total of 450 environmental swabs from 30 transportation vehicles were tested using qPCR. Five out of 30 vehicles (16.67%) tested positive in at least one of the following areas: cargo area or sidecar walls (6.67%), cargo area or sidecar floors (6.67%), hauling personnel’s hands (6.67%), steering wheel or handlebars (3.33%), gear shift levers (3.33%), floor mats or footpegs (3.33%), dashboards (3.33%), door handles or sidecar gate bolts (3.33%), tyres/wheels (3.33%), fenders (3.33%), and hauling personnel’s footwear (3.33%). All investigated risk factors were analysed and were found to be insignificant, including the frequency of swine transportation, frequency of cleaning, cleaning materials used, disinfection practices, the number of pigs transported, and whether hauliers owned pigs (*P* > 0.05). This study illuminates the environmental contamination patterns of ASFV in swine transport vehicles, underscoring the need for targeted biosecurity protocols and more effective vehicle disinfection systems to reduce the risk of ASF disease transmission.

African swine fever (ASF) is a highly contagious viral disease that leads to major morbidity and mortality in swine of all breeds and ages. The disease often proves fatal, with reported mortality rates as high as 100%. There has been a continuous threat to the swine industry from ASF in Africa, Europe, and recently Asia – specifically in China and the Philippines. The Bureau of Animal Industry (BAI) reported the first case of ASF in the Philippines in July 2019; it surfaced at a local farm in Rizal province. Since then, all regions of the Philippines have been affected, including 73 provinces, 965 cities and municipalities, and 5 324 barangays, which denote the smallest territorial and administrative districts in the country’s local government setup. The ASF epidemic led to a 24.1% decrease in the Philippine swine inventory due to direct disease effects and preventive culling. Before the outbreak, the Philippines stood as the 7^th^ largest global pork producer with a volume of 1.64 million metric tons, and it was the 9^th^ ranked country in domestic pork consumption, consuming 1.95 million metric tons (USDA 2019).

The ASF virus (ASFV) is a large, enveloped virus that belongs to the *Asfarviridae* family. It can be transmitted directly and indirectly through infected swine, their products, or vectors, particularly *Ornithodoros* ticks (WOAH 2024). ASFV is a genetically complex, double-stranded DNA virus that carries a range of genes responsible for virulence, immune evasion, and modulation of cellular processes (Tulman and Rock 2001). The ASFV can linger on footwear, clothing, equipment, and pork products (WOAH 2024). The virus can endure extreme conditions such as desiccation, putrefaction, and a wide range of pH levels and temperatures. Owing to these characteristics, environmental contamination of ASFV through secretions and excretions from infected pigs can swiftly spread the disease to susceptible pigs (Mazur-Panasiuk et al. 2019). Indirect transmission through contaminated fomites such as feeds, pens, vehicles, and even veterinarians is a major route for introducing the disease into ASF-free areas (Mur et al. 2012).

Swine transport vehicles have been identified as a significant risk factor for the contamination and spread of the ASFV. A risk assessment of transport-associated routes for the ASFV introduction in Europe revealed that trucks returning from trips posed the highest risk of ASF transmission, surpassing even waste from international ships and planes (Mur et al. 2012). In Russia, contaminated vehicles were found to account for 63.1% of secondary ASF spread, followed by direct contact with infected pigs or their pens (33.3%), and the introduction of new pigs (5.6%) (Khomenko et al. 2013). Similarly, the risk of ASF transmission associated with transport vehicles in domestic pig transportation has been underlined (EFSA 2014). Risk evaluations in other nations, like China and the Netherlands, have also identified transport vehicles as a high-risk factor for the ASF spread (Galindo and Alonso 2017).

Risk assessments across various countries have consistently established that animal transport vehicles present a high risk of the ASF transmission to susceptible regions. However, limited published information is available regarding specific processes for effective cleaning and disinfection of livestock trucks known to be contaminated with the ASFV (Neumann et al. 2021). This study was conducted to examine the patterns of swine transport vehicle contamination, to formulate more targeted biosecurity measures. These could guide relevant authorities in drafting national and local government policies for the mitigation of the ASFV incursion and spread through swine transport vehicles.

## MATERIAL AND METHODS

All procedures performed in this study were approved by the BAI, Department of Agriculture, Philippines, assigned with Animal Research Clearance No. 2022-0034, and the Institutional Animal Care and Use Committee at the College of Veterinary Medicine, University of the Philippines Los Baños (UPLB) with the assigned Protocol No. UPLB-2022-029.

### Study design

This cross-sectional study aims to detect the eDNA of the ASFV in swine transport vehicles. It includes 30 swine transport vehicles, such as trucks and tricycles, that were transporting pigs to slaughterhouses from September 2023 to March 2024. The study sites were identified through purposive sampling. Consent for sample collection was secured from the local government units, private slaughterhouse owners, and the operators of the swine transport vehicles. A questionnaire was distributed to these operators to gather information on the pigs’ province of origin, the frequency of swine transportation per week, cleaning frequency and materials used, disinfection practices, the number of pigs transported, and whether the operators owned any pigs.

### ASFV eDNA sampling

Fifteen distinct sample collection sites were identified on swine transport vehicles and personnel. As previously reported, environmental samples were gathered with minor alterations (Lopez-Lorenzo et al. 2019; Kosowska et al. 2021; Gebhardt et al. 2022). A sterile polyurethane sponge measuring 10 × 7.5 × 4 cm (L × W × H), and hydrated with 10 ml of Normal Saline Solution (NSS), was used for environmental swabbing. Table 1 outlines the swabbing protocol for each location on the swine transport vehicles. A fresh sterile glove was worn for each sample collection. After swabbing, individual environmental swab samples were placed in sterile ziplock bags and stored in a cooler for transport. In the laboratory, the environmental swab samples were rehydrated with 10 ml of a 5% v/v penicillin-streptomycin (Biolab Co., Samut Prakan, Thailand) solution in NSS. Samples were then manually massaged to extract the liquid, and the liquid samples were transferred to a 15 ml graduated conical centrifuge tube and stored at –80 °C. The sample preparation protocols took place within 24 h of environmental swab collection.

**Table 1:** Environmental swab collection for swine transport vehicles and personnel

ASFV eDNA swab samples	Swabbing protocols
Swine transport vehicles	
Steering wheel (trucks and jeeps) or handlebar (tricycle)	whole surface
Gear shift lever	top to bottom in a zigzag pattern
Handbrake or rear brake pedal	top to bottom or side to side in a zigzag pattern
Pedals (trucks and jeeps) or clutch and brake lever (tricycle)	50% of the surface
Floor mats (trucks and jeeps) or footpeg (tricycle)	side to side in a zigzag pattern
Dashboard	side to side in a zigzag pattern
Passenger seat	side to side in a zigzag pattern
Door handle (trucks and jeeps) or sidecar gate bolt (tricycle)	whole surface
Cargo area walls (trucks and jeeps) or sidecar walls (tricycle)	four different sites per wall at 30 **×** 30 cm^2^ per site
Cargo area floor (trucks and jeeps) or sidecar floor (tricycle)	four corners and the centre of the floor at 30 **×** 30 cm^2^ per site
Tyre/wheels	50% of the surface and tread of tyres
Fender	50% of the surface
	
Swine hauling personnel	
Hands	dorsal and ventral surfaces of the hands and fingers, and the ventral surface of the fingernails
Workwear	front and back of the thorax, sleeves preferably from the elbow to the wrist, and pants preferably from the knee to the ankle
Boots/shoes/slippers	dorsal and ventral parts in a zigzag pattern

### DNA extraction

Approximately 140 μl aliquots of each sample were placed in 2-ml microcentrifuge tubes and processed for nucleic acid extraction using commercial kits (QIAamp Viral RNA Mini Kit, Qiagen, West Sussex, UK) according to the manufacturer’s instructions. After the DNA extraction, an exogenous internal PCR positive control (VetMax^TM^ Xeno Internal Positive Control DNA) was added to the extracted samples. Five samples, each consisting of 10 μl of the extracted DNA, were pooled in a 2-ml centrifuge tube. In total, three pooled samples per swine transport vehicle (*n* = 15 areas of the vehicle) were tested. All samples were processed in duplicate.

### ASFV qPCR analysis

The environmental swab samples were analysed using qPCR, targeting the p72 DNA region using commercial qPCR kits (VetMax^TM^ African Swine Fever Detection Kit). Positive and negative controls provided in the kit were used as references. Cycle threshold (Ct) values of ≤45 cycles were considered positive, while Ct values greater than 45 cycles were considered negative, following the manufacturer’s guidelines. Positive pooled samples were tested individually.

### Data analysis

The association between vehicle contamination and the collected vehicle information was tested using Chi-square for categorical values, and the Fisher exact test and odds ratio for two-by-two tables. OpenEpi software v3.01 was used for statistical analysis (Dean et al. 2013). A two-tailed test was applied, and a *P*-value <0.05 was considered statistically significant.

## RESULTS

### ASFV eDNA positivity rate in swine transport vehicles

A total of 450 environmental samples were collected from 30 swine transport vehicles across various slaughterhouses in select regions of the Philippines. The study identified two types of vehicles, specifically trucks and tricycles. Most vehicles delivering pigs to the slaughterhouses were trucks (19 out of 30), with the remainder being tricycles (11 out of 30). Among the 30 vehicles examined, the ASFV eDNA was detected in five vehicles, resulting in a positivity rate of 16.67% (Table 2). Three trucks (13.64%) and two tricycles (25%) tested positive for the ASFV qPCR. These vehicles each had at least one positive result out of 15 sampled areas on the vehicle (Table 3). The pigs transported by the ASF-positive vehicles originated from various locations, including San Jose in Batangas, Capas in Tarlac, General Trias in Cavite, Candelaria in Quezon, and Alaminos in Laguna.

**Table 2:** ASFV eDNA positivity rate using qPCR

ASFV eDNA swab samples	No. of positive samples	Average cycle threshold (Ct) value	Positivity rate (%)
Swine transport vehicles			
SW – steering wheel (trucks) or handlebar (tricycle)	1	38.1	3.33
GS – gear shift lever	1	29.0	3.33
HB – handbrake (trucks) or ear brake pedal (tricycle)	0	undefined	0
P – pedals (trucks) or clutch and brake lever (tricycle)	0	undefined	0
FM – floor mats (trucks) or footpeg (tricycle)	1	36.0	3.33
DB – dashboard	1	33.6	3.33
PS – passenger seat	0	undefined	0
DH – door handle (trucks) or idecar gate bolt (tricycle)	1	33.5	3.33
CAW – cargo area walls (trucks) or sidecar (tricycle) walls	2	35.4/40.2	6.67
CAF – cargo area floor (trucks) or sidecar(tricycle) floor	2	33.8/35.9	6.67
T – tyre/wheels	1	36.4	3.33
F – fender	1	38.0	3.33
			
Swine hauling personnel			
H – hands	2	35.7/42.4	6.67
WW – workwear	0	undefined	0
B – boots/shoes/slippers	1	38.5	3.33
			
Total positive and positivity rate	14	–	3.11

**Table 3:** Analysis of management practices of swine transport vehicles and hauliers

Vehicle No.	No. of positive areas	Positivity rate (%)	Origin of pigs	Frequency of transportation per week	Materials for cleaning/ disinfection	Frequency of cleaning and disinfection	Pig owners
1	0	0	Alabat, Quezon	3–4 times	detergent/none	after pig delivery	no
2	2	13.33	San Jose, Batangas	irregular	detergent/none	after pig delivery	no
3	0	0	San Jose, Batangas	daily	detergent/unknown powder	before loading	no
4	1	6.67	Capas, Tarlac	daily	detergent/none	daily	no
5	0	0	Ibaan, Batangas	3–4 times	detergent/none	before and after loading	no
6	2	13.33	General Trias, Cavite	daily	detergent/chlorine	before and after loading	no
7	0	0	Gerona, Tarlac	daily	detergent/none	daily	no
8	0	0	Ibaan, Batangas	3–4 times	detergent/none	twice a week	no
9	0	0	Candelaria, Quezon	3–4 times	detergent/alcohol	every pig delivery	yes
10	0	0	Candelaria, Quezon	1–2 times	detergent/none	every pig delivery	no
11	0	0	Candelaria, Quezon	daily	water/none	daily	no
12	0	0	Candelaria, Quezon	daily	water/none	daily	no
13	0	0	Candelaria, Quezon	daily	detergent/none	daily	no
14	0	0	Candelaria, Quezon	daily	detergent/none	daily	no
15	0	0	Candelaria, Quezon	daily	water/none	daily	no
16	0	0	Candelaria, Quezon	daily	water/none	daily	no
17	0	0	Candelaria, Quezon	daily	water/none	daily	no
18	1	6.67	Candelaria, Quezon	daily	water/none	daily	no
19	0	0	Tiaong, Quezon	5–6 times	detergent/none	before and after loading	no
20	0	0	Tiaong, Quezon	1–2 times	detergent/chlorine	after pig delivery	no
21	0	0	Palayan, Nueva Ecija	3–4 times	detergent/none	after pig delivery	no
22	0	0	Calaca City, Batangas	daily	detergent/none	after pig delivery	yes
23	8	53.33	Alaminos, Laguna	3–4 times	water/none	after pig delivery	no
24	0	0	Sta. Maria, Laguna	3–4 times	dishwashing liquid/none	after pig delivery	no
25	0	0	Sta. Cruz, Laguna	3– 4 times	water/none	every pig delivery	no
26	0	0	Sariaya, Quezon	1–2 times	dishwashing liquid/chlorine	twice a week	yes
27	0	0	Rosario, Batangas	1–2 times	dishwashing liquid/chlorine	after pig delivery	no
28	0	0	Sta. Maria, Laguna	3–4 times	dishwashing liquid/chlorine	after pig delivery	no
29	0	0	Candelaria, Quezon	3– 4 times	dishwashing liquid/chlorine	every pig delivery	no
30	0	0	Trese Martirez, Cavite	daily	dishwashing liquid/none	daily	no

### ASFV eDNA contamination patterns in swine transport vehicles

Eleven out of 15 areas analysed using surface swabs on the vehicles tested positive for ASFV eDNA (Table 2). Positivity rates of 6.67% were observed in the cargo area walls (trucks) or sidecar walls (tricycles), cargo area floors (trucks) or sidecar floors (tricycles), and hands of hauling personnel. By contrast, areas like the steering wheel (trucks) or handlebar (tricycles), gear shift lever, floor mats (trucks) or footpeg (tricycles), dashboard, door handle (trucks) or sidecar gate bolt (tricycles), tyres/wheels, fenders, and footwear of hauling personnel had a positivity rate of 3.33%. No ASFV eDNA was detected on the handbrake (trucks) or rear brake pedal (tricycles), pedals (trucks) or clutch and brake levers (tricycles), passenger seat, or workwear.

Among all the swine transport vehicles, 3.33% had the highest number of positive areas with a positivity rate of 53.33% (8 out of 15 areas on the vehicles) (Table 3). Similarly, 6.67% of the vehicles had 13.33% of their areas test positive (2 out of 15 areas). Another 6.67% of the vehicles held a positivity rate of 6.67% (1 out of 15 areas).

### Analysis of management practices of swine transport vehicles

The distribution of positive vehicles across various management practices was examined (Table 3). The frequency of pig transportation and cleaning practices indicates that daily transportation and cleaning were the most common (3/5), while some cleaned and delivered pigs three to four times per week (1/5), or at irregular intervals (1/5). Vehicles using detergent for cleaning were more common (3/5) than those using only water (2/5). Disinfection practices among positive vehicles were categorised as follows: hauliers administering disinfectant (2/5), disinfection at the pig farm source or checkpoints (1/5), and no disinfection (2/5). The number of pigs transported by the positive vehicles was classified as 1–10 (2/5), 31–40 (2/5), and more than 40 (1/5). Lastly, none of the hauliers of positive vehicles owned pigs themselves.

### Epidemiological measures of association

No association was detected between the type of vehicle used and the likelihood of acquiring ASFV (*P* = 0.864 4). The Chi-square test did not reveal any significant correlation between vehicle positivity and the delivery slaughterhouses (*P* = 0.307 2). Similarly, no significant difference was found regarding the distribution of ASFV in vehicles based on the province of origin of the delivered pigs (*P* = 0.456 2). Various factors analysed using the Chi-square, Fisher’s exact test, and odds ratio were deemed insignificant. These included swine transportation frequency per week (*P* = 0.141 0), cleaning frequency per week (*P* = 0.205 8), the type of cleaning materials used (*P* = 0.506 5), disinfection practices (*P* = 0.944 0), the number of pigs transported (*P* = 0.248 7), as well as whether hauliers owned the pigs or not (*P* = 0.566 5) (Table 4 and 5). All *P*-values were above the significance level of 0.05.

**Table 4:** Analysis of epidemiological measures using Chi-square analysis

Factors	Categories	Chi-square	*df*	*P*-value
Different vehicle and hauler sites for collection	SW, GS, HB, P, FM, DB, PS, DH, CAW, CAF, T, F, H, WW, B	7.667	14	0.905 9
Province of origin of pigs delivered to slaughterhouse	Laguna, Batangas, Tarlac, Nueva Ecija, Quezon, Cavite	4.68	5	0.456 2
Frequency of swine transportation in a week	daily, 5–6 times, 3–4 times, 1–2 times, irregular	6.905	4	0.141 0
Frequency of vehicle cleaning in a week	daily, 5–6 times, 3–4 times, 1–2 times, irregular	5.912	4	0.205 8
Disinfection practices	yes, none, at farms/checkpoints	0.115 3	2	0.944 0
Number of pigs transported	1–10, 11–20, 21–30, 31–40, >40	5.4	4	0.248 7
Slaughterhouse delivered	Valenzuela City, Quezon City, Caloocan City, Candelaria, Los Baños	4.812	4	0.307 2

B = boots/shoes/slippers; CAF = cargo area floor (trucks) or sidecar (tricycle) floor; CAW = cargo area walls (trucks) or sidecar (tricycle) walls; DB = dashboard; DH = door handle (trucks) or sidecar gate bolt (tricycle); F = fender; FM = floor mats (trucks) or footpeg (tricycle); GS = gear shift lever; H = hands; HB = handbrake (trucks) or rear brake pedal (tricycle); P = pedals (trucks) or clutch and brake lever (tricycle); PS = passenger seat; SW = steering wheel (trucks) or handlebar (tricycle); T = tyre/wheels; WW = workwear

**Table 5:** Analysis of epidemiological measures using Fisher’s exact mid-*P* analysis

Factors	Categories	*P*-value (2-tail)	Odds ratio	Confidence interval
Cleaning materials	water, detergent	0.506 5	0.487 1	0.058 35, 4.917
Type of vehicles	truck, tricycles	0.864 4	0.848 6	0.106 7, 8.276
Haulers owning pigs	yes, none	0.566 5	0	0.0, 9.31

## DISCUSSION

ASF has consistently devastated the Philippine swine industry, resulting in substantial economic loss (Cooper et al. 2022). The disease’s spread through direct contact with infected pigs and indirect encounters via contaminated fomites, raw meat, or arthropod vectors has been widely studied to control transmission. In the absence of effective treatments and vaccines for ASF, the illness continues as a severe threat to food security and the economy. Environmental contamination is acknowledged as a key method of virus dispersion due to the virus’s resilience under varying ecological conditions (Brown and Bevins 2018). Many studies have emphasised the high-risk mechanical transmission of ASF through live pig transport using vehicles examined with risk assessments and mathematical models (Bellini et al. 2021; Cheng and Ward 2022; Hsu et al. 2023). Despite this, concrete information investigating specific areas of swine transport vehicles at high risk for ASF contamination is scarce. There is also a lack of studies exploring the management practices of eDNA-positive swine transport vehicles. This research sought to examine the eDNA contamination patterns of ASFV through surface sampling of 15 different areas of swine transport vehicles and link these findings to the management practices of swine hauliers. Recognising the patterns of ASF eDNA contamination will support the development of more targeted biosecurity measures to prevent ASFV’s spread through swine transport vehicles.

The detection of ASFV eDNA in 16.67% of swine transport vehicles strongly suggests the potential for virus transmission via the movement of pigs. These findings align with other studies that underscore the risk of transporting live pigs and their vehicles. Cheng and Ward (2022) identified live pig transport and vehicles as significant risk factors for ASFV spread in China. The transportation of infected pigs from finisher farms to abattoirs can contaminate the vehicle, allowing ASFV to transmit to the subsequent batches of pigs, feed, or farm equipment (Cheng and Ward 2022). A risk assessment conducted by veterinary experts in the Philippine swine industry, using conjoint and SWOT analysis, also identified contaminated vehicles as a primary risk factor for commercial farms (Hsu et al. 2023). Similarly, vehicle contamination was found to be a major risk factor in European epidemiological studies (Bellini et al. 2021).

Different areas of a vehicle frequently come into direct contact with farms, animals, and humans. Cargo floors and walls of these vehicles show more positive results for the ASF virus than other parts that tested positive. This outcome can be connected to the cargo regions of swine transport vehicles, which are often in direct contact with potential contamination sources such as infected live animals. The excretions of any infected animals can easily contaminate these surfaces. The tyres, wheels, and fenders could acquire the virus upon entering an infected farm or other contaminated facilities within the value chain. Gebhardt et al.’s (2022) study on environmental sampling within a feed manufacturing and swine production system also recognises the presence of ASFV in vehicles, where both the cargo area and wheels tested positive for the ASF virus.

ASFV eDNA was detected on the hauliers’ hands and footwear. Clothing and footwear are considered fomites playing a crucial role in spreading the virus (WOAH 2024). A study in Latvia showed that human entry into infected farms was related to secondary outbreaks of ASFV (Olesen et al. 2017). Environmental contamination of vehicle parts without direct contact with infected animals or farms can be attributed to human activities. This presents an environmental risk factor for ASF, capable of transmitting the disease over long distances as documented by Bergmann et al. (2022). Some human activities contributing to the spread of ASF include manure management, pest control, feed storage, cadaver management, outdoor pig keeping, and pig transportation (Bergmann et al. 2022). Contaminated hands of hauliers can touch the truck steering wheel, dashboard, door handle, or tricycle handlebar and sidecar gate bolt. Contaminated footwear can directly contact the gear shift lever and floor mats of trucks or the footpeg of a tricycle. These interactions can readily transfer the virus to other parts of the vehicle. Moreover, the Philippines, a tropical country, sees a higher risk of ASFV during the rainy season, as floods can more readily spread the virus in areas where pigs were culled and buried. It is suspected that floodwater reintroduces the ASFV into farm environments, contaminating footwear, transportation trucks, and other fomites, leading to a spike in ASF cases every rainy season.

Swine transport vehicles bring pigs from farms to abattoirs. Many of these vehicles, originating from different locations, converge at abattoirs for pig delivery. Due to the archipelagic geography of the Philippines, the swine market is highly fragmented. The harvested pigs are primarily marketed by unorganised third-party middlemen and transported via trucks. The interisland shipment of pigs is facilitated through roll-on roll-off cargo ships, where trucks from multiple hauliers often intermingle within the ship’s cargo area. This common contact area for swine transport vehicles can serve as a medium for viral spread. Fasina et al. (2012) identified slaughterhouses as the highest risk area for ASF infection because pig farmers often transport sick pigs for immediate slaughter at the nearest abattoir. Tissues and fluids from infected animals can contaminate the slaughterhouse environment, leading to the subsequent spread of the virus to swine transport vehicles (Fasina et al. 2012). Although 73.33% of the swine transport vehicles included in this study were cleaned and disinfected, the African swine fever virus (ASFV) was still detected. This suggests that the cleaning methods and disinfectants used may not have been effective in eliminating environmental contamination, leading to a positive test result. It was found that most hauliers rely on regular detergent and dishwashing liquid for routine cleaning. For vehicle disinfection, alcohol and chlorine were more commonly used; however, ASFV is resistant to these types of disinfectants. A comprehensive guide on the proper cleaning and disinfection of vehicles should be incorporated into official guidelines to control and prevent the spread of ASF.

Biosecurity measures, subsidised by the government, can be implemented in abattoirs to reduce the transmission of the ASF virus from abattoirs to swine farms. The National Meat Inspection Service (NMIS) has issued Administrative Order (AO) No. 32 series of 2002 Section 2d, mandating that vehicles must pass through a wheel bath upon entering the slaughterhouse (NMIS 2002). However, considering the detection of the ASF virus on vehicle wheels, despite equipping all slaughterhouses with wheel baths, their effectiveness requires re-evaluation. To optimise the usage of wheel baths, the disinfectant must be replenished regularly, the entry of rainwater should be prevented, and the bath should be kept clear of mud and manure while adhering to appropriate soaking durations (Liu et al. 2021).

Cross-regional and provincial transportation of pigs is a common practice that can introduce infected live pigs and contaminated vehicles into ASF-free zones (Bellini et al. 2021). Implementing strategically placed wheel baths and foot baths along major animal transportation routes, seaports, and provincial entry points could significantly reduce the spread of ASFV via vehicles and human activity. It was also observed that most swine transport vehicles in the study were cleaned at the loading dock after moving the pigs into the slaughterhouses’ resting pens. This practice complies with NMIS AO No. 32 series of 2022 Section 2d, stipulating that all vehicles must be properly cleaned and disinfected before leaving the slaughterhouse (NMIS 2002). However, cleaning at the docking area may promote environmental contamination if appropriate disinfection is not performed. Establishing specific areas within the slaughterhouse as cleaning zones, equipped with proper drainage and controlled water flow, could mitigate this risk. In these areas, cleaning and disinfection could be conducted according to prescribed standard procedures. Proper cleaning and disinfection of vehicles before and after pig transportation would drastically reduce the risk of spreading ASFV (Bremang et al. 2022).

In conclusion, this study has unveiled the patterns of ASF eDNA contamination in swine transport vehicles and its potential role in transmitting the virus. The findings underscore the ease with which ASFV can spread from one area to another via vehicle movement. This highlights the need for additional biosecurity measures, such as the establishment of more disinfection stations and the application of stricter protocols on farms and slaughterhouses. The data pinpoints the areas of swine transport vehicles that are at greatest risk of contamination, underscoring the importance of targeted cleaning and disinfection protocols. The study shows that the cabin areas of swine transport vehicles, where current disinfection practices are often inadequate, have a high frequency of ASF eDNA detection. This accentuates the need for technologies capable of thoroughly disinfecting these areas without causing damage to the vehicles or hazards for handlers. Although no correlation was found between vehicle management and movement, or the behaviours of hauling personnel, increasing the sample size and/or sampling areas could clarify these relationships and identify trends. Farmers and local government units are reluctant to participate in such studies, as the ASFV detection can impact a region’s official zoning status in the Philippines, affecting pig movement and trade. Further research is recommended to develop effective, safe decontamination systems for swine transport vehicles, reducing the risk of ASF disease transmission between countries and regions. While the viral DNA on a surface signifies contamination, eDNA detection does not confirm that the contaminated surface can cause infection directly. Therefore, more studies are needed to confirm infectivity and determine the infectious dose from ASF eDNA.
